# News and Events of the Week

**Published:** 1911-03-11

**Authors:** 


### News and Events of the Week.

The annual general meeting of the Medical Graduates' College and Polyclinic will be held at 22 Chenies Street, London, W.C., on Friday, March 31, at 5.15 p.m.

"Open Air to Everywhere" is the title of a new road map issued by the London General Omnibus Co., Ltd., 9 Grosvenor Road, Westminster, and which shows all the 'bus routes for the Metropolitan and suburban area.

A Bill to regulate the qualifications of trained nurses and to provide for their registration has been presented to Parliament by Mr. Munro Ferguson, and is supported by Sir James Gibson, Sir Luke White, Mr. Rainy, Dr. Addison, Mr. Ramsay Macdonald, and Mr. Field. Particulars of the Bill appear in this week's *Nursing Mirror*. There is no likelihood that the Bill will be passed this session.

The Prudential Assurance Co., Ltd.—This well-known Assurance Company is likely to become one of the most important, if not the most important, in the Kingdom in consequence of the proposed State insurance scheme. It is a very strong association, and the 62nd annual report shows that its position is every day improving. The bonuses paid to policy holders vary from 5 to 20 per cent., according to the amount of the premiums paid. Medical men who are not yet assured cannot do better than write for the prospectus and outline policies of the Prudential.

The staff of the Post-graduate College, West London Hospital, will hold a conversazione at the Hospital on Wednesday, March 15, at 8.30 p.m.

By the kindness of the Lord Mayor of London, a great public meeting in aid of the National Society for the Prevention of Cruelty to Children will be held at the Mansion House, on Monday, May 29, at 3.30 p.m. At the invitation of the Branch Committee, there will be an Autumnal Congress of the same Society in Dublin in October.

Although the question of "diet" has received considerable attention at many of the foreign Spas, British health resorts have to a large extent neglected this important part of the cure. "A special bath diet" for those undergoing the cure has just been adopted by the Bath branch of the British Medical Association, and the principal hotels and boarding-houses have cordially co-operated by arranging for this diet to be always available on their menu. The list has been made as full as possible, so as to offer a reasonable choice, and it is so arranged that any medical man may make such modifications as he thinks desirable to suit individual cases. Physicians have frequently complained of the neglect of dieting at British Spas, and it is believed that the adoption of the special diet will entirely remove this objection as far as Bath is concerned, and for those patients who require special dieting will form a very valuable adjunct to the mineral water cure.

At the Central London Throat and Ear Hospital, Gray's Inn Road, W.C., the following lectures will be given on Tuesday, March 14, at 3.45 p.m. : "Larynx," Dr. Andrew Wylie; Friday, March 17, 3.45 p.m. : "Larynx," Dr. Andrew Wylie.

Professor Hahn, of the University of Berlin, has discovered in the residues of gas-mantles a new chemical compound, which he has named *mesothorium.* The bromate of this chemical substance develops the same radio-active power as radium itself. The annual production of the bromate in Germany amounts to about 10 grammes, as against about an equal production of radium in the whole world.

"The Diagnosis of Smallpox," by Dr. T. F. Ricketts, medical superintendent of the Smallpox Hospitals and of the River Ambulance Service of the Metropolitan Asylums Board, has been written with the co-operation of the Metropolitan Asylums Board. The most noteworthy difference between its teaching and that of previous writers on the subject resides in the importance attributed to the distribution of the eruption : a diagnostic criterion which has been lifted from a subordinate to a leading position. It is the first medical work to be illustrated freely by means of colour-photographs taken from life, all the colour-plates being from triple negatives obtained by the Sanger-Shepherd process of colour photography, and is published by Cassell and Company.

The following appointments have been confirmed at the Manchester Royal Infirmary : Cancer Research Officer : Mr. C. B. Marshall, M.B., Ch.B. Vict. ; Senior House Surgeons : Mr. E. L. Horsburgh, M.B., B.S.Lond. ; Mr. F. H. Diggle, M.B., Ch.B. Vict., M.R.C.S., L.R.C.P. ; House Physician : Mr. R. F. Higgin, M.R.C.S., L.R.C.P. ; Assistant Director of the Clinical Laboratory : Mr. C. E. Lea, M.D., Ch.B. Vict. ; Sixth Anæsthetist : Mr. G. Jefferson, M.B., B.S. Lond., M.R.C.S., L.R.C.P.

The following are the arrangements for next week at the Medical Graduates' College and Polyclinic, 22 Chenies Street, W.C. : Monday, March 13, at 4 p.m., Dr. George Pernet, Clinique (Skin) ; at 5.15 p.m., Dr. J. Blümfield, "Anæsthetics in Abdominal Operations." Tuesday, at 4 p.m., Dr. James Collier, Clinique (Med.) ; at 5.15 p.m., Mr. Somerville Hastings, "Chronic Suppuration of the Antrum." Wednesday, at 4 p.m., Mr. Chas. Ryall, Clinique (Surg.) ; at 5.15 p.m., Dr. F. J. Wethered, "Asthma." Thursday, at 4 p.m., Mr. G. B. White, Clinique (Surg.) ; at 5.15 p.m., Dr. D. Forsyth, "Diagnostic Difficulties in Children." Friday, at 4 p.m., Mr. M. S. Mayou, Clinique (Eye).

The following are the arrangements for next week at West London Postgraduate College, West London Hospital, Hammersmith Road : March 13 and 16, at 10 a.m., demonstration by Surgical Registrar; March 15, at 10 a.m., gynæcological demonstration; March 14, at 11.30 a.m., demonstration of minor operations; March 13, at 12 noon, pathological demonstration; March 16, at 12.15 p.m., lecture, Dr. Grainger Stewart, "Practical Medicine." Lectures, at 5 p.m. : March 13, Mr. Swinford Edwards, "Urinary Surgery"; March 14, Mr. Bidwell, "Minor Surgery"; March 15, Dr. Robert Jones, "The question whether there is an insane temperament"; March 16, Mr. Baldwin, "Practical Surgery, Lecture VI." ; March 17, Dr. Abraham, "Cases of Skin Disease."

The third Congress of the International Society of Surgery will be held in Brussels from September 26 to September 30, 1911. On the occasion of the meeting there will be held (1) an exhibition of preparations and documents relative to the study and treatment of fractures, to which all doctors will be admitted on making application to the general secretary, subject to ratification by the National Committee; (2) an exhibtion of surgical apparatus and instruments. The meetings of the society will be public, but only members will be allowed to take part in the discussions. Three questions are open for debate—(*a*) Pleuro-pulmonary surgery, upon which papers will be read by MM. Garré of Bonn, Gaudier of Lille, Girard of Geneva, Lenormand of Paris, Ferguson of Chicago, Van Stockum of Rotterdam, Sauerbruch and Friedrich of Marburg; (*b*) Colitis, papers by MM. Sonnerburg of Berlin, Segond of Paris, Gibson of New York, and D'Arcy Power of London; and (*c*) Pancreatitis, papers by MM. Michel of Nancy, Korte of Berlin, Giordano of Venice. For information on all points application should be made to the general secretary, M. le Professeur Depage, 75 Avenue Louise, Brussels, Belgium.

During the next month or two Messrs. Longmans, Green, and Co. will publish the Life and Letters of Sir John Hall, M.D., K.C.B., the Principal Medical Officer of the Crimea during the great campaign. The unique feature of this publication is that it is the first biography of a distinguished Englishman written by a well-known Hindu writer. The work is by Mr S. M. Mitra, author of "Indian Problems," "Hindupore," &c. Rear-Admiral Sir Massie Blomfield, who served throughout the Crimean campaign, has written an introduction. The book deals with Sir John Hall's career in Flanders, the West Indies, Spain, South Africa (including the Kafir War of 1852), India, and the Crimean campaign. It will contain several illustrations, including one of the wounded at Waterloo, whom as hospital assistant Dr Hall attended.

A new society, the Institut Français d'Anthropologie, has just been founded with headquarters in the Paris Museum of Natural History (in the Laboratory of Anthropology). The new institute proposes to bring together anthropologists, palæontologists, zoologists, anatomists, histologists, biologists, physiologists, psychologists, archæologists, geologists, ethnographers, and travellers, with the object of utilising their work for the fuller investigation of anthropology among the ancients and uncivilised peoples. The society will be limited to 50 members. The committee as constituted at present consists of the president, M. Salomon Reinach; vice-president, M. Marcellin Boule; general secretary, M. Lapicque; treasurer, M. Hubert; librarian, M. Rivet. Councillors : M. Dürkheim, Professor at the Sorbonne; M. Grandier, Member of the Academy of Science; M. Meillet, Professor at the Collège de France; and M. Verneau, Professor at the Museum.

Messrs. A. and C. Black are about to publish an English translation of the fifth German edition of Prof. Kocher, the famous Bernese surgeon's text-book of Operative Surgery, which will be the third English edition. The whole book has been thoroughly revised and rearranged. Many of the sections have been rewritten, while the additions which have been made both to the text and illustrations are such as to make the work now cover the whole field of operative surgery.

